# 单形性嗜上皮性肠T细胞淋巴瘤的诊疗现状及进展

**DOI:** 10.3760/cma.j.cn121090-20251128-00557

**Published:** 2026-05

**Authors:** 帅 苏, 毅 肖

**Affiliations:** 华中科技大学同济医学院附属同济医院血液内科，武汉 430030 Department of Hematology, Tongji Hospital, Tongji Medical College, Huazhong University of Science and Technology, Wuhan 430030, China

## Abstract

单形性嗜上皮性肠T细胞淋巴瘤（MEITL）是一类罕见且具有高度侵袭性的肠道T细胞淋巴瘤，近年来随着高通量测序技术的应用，对该病的认识越来越全面，世界卫生组织已将其明确为独立疾病实体。然而，由于其罕见性，MEITL在临床诊断和治疗方面仍面临巨大挑战，且患者预后较差。本文在系统梳理近年文献的基础上，综述MEITL基于病理及分子生物学的诊断标准以及以手术、化疗及造血干细胞移植为基础的综合治疗策略。同时，结合现有的临床和转化研究，探索表观遗传药物、JAK/STAT通路抑制剂及其他新兴策略在MEITL中的应用潜力。本文旨在为临床梳理MEITL的诊疗现状及进展，并为未来前瞻性、多中心临床研究的设计提供参考。

## Abstract (en)

Monomorphic epitheliotropic intestinal T-cell lymphoma（MEITL）is a rare and highly aggressive type of intestinal T-cell lymphoma. With the application of high-throughput sequencing technologies in recent years, the understanding of this entity has become increasingly comprehensive. The World Health Organization classification of lymphoma now recognizes MEITL as a distinct disease entity. However, the extreme rarity of MEITL poses substantial challenges for clinical diagnosis and management, and patient outcomes remain poor. Based on a systematic review of recent literature, this article summarizes the diagnostic criteria of MEITL, integrating pathology and molecular biology; outlines current multimodal treatment approaches centered on surgery, chemotherapy, and hematopoietic stem cell transplantation; and reviews available clinical and translational data to explore the potential role of epigenetic agents, JAK/STAT pathway inhibitors, and other emerging therapeutic strategies in MEITL. This article summarizes the current status and recent advances in the diagnostic and therapeutic strategies for MEITL in clinical practice and provides a reference for the design of future prospective, multicenter clinical studies.

单形性嗜上皮性肠T细胞淋巴瘤（MEITL）是一类罕见且侵袭性极高的外周T细胞淋巴瘤，曾被称为肠病相关T细胞淋巴瘤（EATL）Ⅱ型，在2016年被世界卫生组织正式更名为MEITL，与经典的EATL相区别[1]。EATL与MEITL在外周T细胞淋巴瘤中的总体发病率不足5％，而在无乳糜泻背景的亚洲，MEITL是最常见的原发肠道T细胞淋巴瘤类型。患者多为中老年人，研究报道其中位发病年龄为60～70岁，性别未见差异[2]。与EATL不同，MEITL患者无乳糜泻病史，其发病机制尚未明确，可能源于小肠固有的上皮内T淋巴细胞克隆性增殖，尤其以γδT细胞最常见[3]。

MEITL常以起病隐匿、进展迅速的病程为特征。多数患者以非特异性消化道症状起病，如腹痛、体重下降、腹泻等。MEITL进展迅速，侵袭性极高。部分患者直至出现并发症（穿孔、出血或肠梗阻）时才被明确诊断。MEITL最常受累部位为小肠，以空肠多见，可累及结肠[4]。未经治疗时患者预后极差。即使接受传统治疗方案，多项研究显示MEITL患者的中位生存期通常不足1年[4]–[6]。为梳理MEITL近年来在诊断与治疗方面的研究现状与进展，本文就其诊断依据、预后评估及治疗探索作一综述，以期为临床实践和后续研究提供参考。

一、诊断标准与进展

（一）临床表现

MEITL通常以非特异性症状起病，常见主诉包括持续或反复的腹痛、腹胀、消瘦和乏力。由于疾病进展迅速，不少患者首发表现即为急腹症。韩国一项单中心研究纳入35例MEITL患者，其中21例患者发生肠穿孔[7]；一项多中心队列中26％的患者因身体虚弱或并发症未能接受任何化疗而仅行紧急手术[8]。以上提示MEITL患者常以危重状态就诊。实验室检查同样缺乏特异性，多数患者在诊断时出现LDH、C反应蛋白升高以及白蛋白降低。由于疾病的罕见性且表现的非特异性，诊断可能会有延迟，需要临床医师警惕。

（二）内镜与影像学检查

MEITL主要累及小肠，上消化道内镜或结肠镜检查受限，胶囊内镜有助于观察小肠病变情况。在既往报道的病例中，肠镜下MEITL可表现为弥漫性黏膜水肿、镶嵌样黏膜粗糙增厚，伴多发浅表溃疡或糜烂，病变区可有狭窄[9]。内镜检查典型病变见附图1A（**扫描本文首页二维码查阅**）。影像学在MEITL的诊断和分期中至关重要。腹部增强CT常见表现为小肠局部壁不均匀增厚或肿块形成，伴肠腔狭窄，有时可见多个不连续肿块和肠套叠；合并穿孔时表现为游离腹腔积气和局部脓肿形成[10]。^18^F-氟代脱氧葡萄糖（FDG）PET-CT对该病具有较高敏感性，MEITL病灶呈现高代谢聚集，SUV值明显增高，反映其高度侵袭性。PET-CT有助于评估病变范围、分期及疗效。

（三）组织病理学

病理检查是确诊MEITL的金标准。通常通过手术切除或内镜活检获得病变组织。病灶多为肠壁局限性肿块或弥漫性浸润，可导致溃疡形成，部分病例为多灶性。镜下MEITL典型形态为单形性的中等大小异型淋巴细胞弥漫浸润肠壁各层，以黏膜层和上皮内浸润最为显著。细胞核呈圆形或不规则，染色质粗颗粒状，胞质较少。上皮亲和性是诊断关键：肿瘤细胞沿小肠上皮成排浸润，形成上皮内淋巴细胞增生样改变（**扫描本文首页二维码查阅附图1**）。与经典EATL不同，MEITL通常缺乏广泛的坏死和明显的炎性背景。然而，约40％的病例可能呈现某些不典型形态，如核异型性、出现散在巨细胞或星空状增殖区域，偶见血管侵犯或灶性坏死。这些病例形态上区别于经典单形性表现，需结合免疫组化和分子检测综合诊断[2]。

（四）免疫组化

MEITL具有特征性的免疫表型，有助于与其他亚型T细胞淋巴瘤鉴别，典型免疫组化结果见附图2（**扫描本文首页二维码查阅**）。肿瘤细胞普遍表达T细胞抗原：CD3强阳性；CD7常保留表达（97％）。CD4和CD5呈阴性（>90％患者缺失）。绝大多数MEITL表达CD8（90％）和CD56（86％）。细胞毒性分子如颗粒酶B、T细胞胞内抗原1和穿孔素阳性，反映其细胞毒性T细胞本质。MEITL不表达CD30，可与大多数EATL和间变性大细胞淋巴瘤明显区分。部分病例可以异常共表达B细胞抗原，但PAX5、BCL6等生发中心B细胞标志物阴性，因此可与B细胞淋巴瘤明显鉴别。Ki-67在MEITL中常高表达（40％～80％），提示高侵袭性特征[2],[10]。此外，小肠上皮淋巴细胞相关的整合素CD103在多数MEITL中阳性，可作为诊断的支持依据。约一半MEITL为T细胞受体（TCR）γδ阳性，这一点与多数病例无CD4/CD8典型分化表型一致[2],[11]。免疫表型分析对于确诊肠道T细胞淋巴瘤的类型至关重要，必要时应做T细胞受体基因重排检测以确认克隆性。

（五）分子生物学

高通量测序技术协助确立了MEITL特征性的分子突变，用于辅助诊断和探索治疗靶点。最具代表性的发现是SET域包含2,组蛋白赖氨酸甲基转移酶（SETD2）基因的失活突变。近年多项研究报道发现SETD2突变或缺失率为70％～97％，可视为MEITL的分子标志物[5],[12]–[14]。SETD2编码组蛋白甲基转移酶，其突变导致组蛋白H3第36位赖氨酸三甲基化缺失。此外，MEITL中最常见的分子异常集中在JAK/STAT信号通路和表观遗传调控相关基因。JAK3和信号转导和转录激活因子5B（STAT5B）是MEITL中高频突变基因，发生率为50％～60％，而EATL常见STAT3和JAK1激活突变，存在明显差异[5],[13],[15]。约三分之一的MEITL患者发生TP53突变，与不典型形态显著相关，提示p53失控可能驱动更具侵袭性的表型[2]。Ras/MAPK通路基因如KRAS/NRAS在部分MEITL病例中也存在典型热点突变[11]。此外，MEITL还表现出以8q24/MYC增益（50％）与9q34.3增益（75％）为代表的拷贝数异常[16]。

综上，MEITL分子发病机制以组蛋白甲基化异常和JAK/STAT信号过度激活为特征，可针对上述基因设计基因检测面板，在可疑病例中寻找支持诊断的突变依据。综合近年相关研究[17]–[20]，MEITL与其他肠道淋巴瘤的鉴别诊断要点详见表1。

**表1:** 单形性嗜上皮性肠T细胞淋巴瘤的鉴别诊断

疾病名称	临床特征	形态学	免疫表型	分子异常	鉴别要点
肠病相关T细胞淋巴瘤	乳糜泻相关；西方多见	多形性大细胞侵袭性浸润、炎性背景	CD3阳性、CD30多为阳性；CD4、CD8、CD56多为阴性	JAK1/STAT3突变多见；>80％见9q34.3扩增/16q12.1缺失	乳糜泻背景；多形性、炎性背景；CD30阳性、CD56阴性
胃肠道惰性T细胞淋巴瘤	病程缓慢；慢性腹痛或腹泻	小细胞表浅浸润	CD4或CD8阳性；CD56阴性	STAT3::JAK2基因融合	惰性病程；浅层浸润；CD56阴性
胃肠道惰性NK细胞淋巴增殖性疾病	自限性病程；无症状或非特异性腹部症状	非典型中等大小表浅浸润	NK细胞表型：CD56、CD16阳性；CD3阴性	JAK3突变	惰性病程；浅层浸润；NK细胞表型
结外NK/T细胞淋巴瘤	侵袭性；肠穿孔常见	多形性细胞侵袭性浸润；血管破坏性生长，坏死显著	CD2、CD56、EBER阳性；CD3阴性	EBV相关；JAK-STAT通路、表观遗传调控基因、TP53、DDX3X突变；6q21缺失	EBER阳性；血管破坏、显著坏死

**注** NK细胞：自然杀伤细胞；EBV：EB病毒；EBER：EBV编码的小RNA

二、预后预测模型

MEITL总体侵袭性强，且早期事件风险突出，不同队列间结局仍存在一定异质性，因此建立可操作的风险分层对治疗决策尤为关键。目前尚无成熟的预后评分系统，临床实践多借用淋巴瘤或外周T细胞淋巴瘤的通用模型进行分层。国际预后指数（IPI）以年龄、血清LDH水平、美国东部肿瘤协作组（ECOG）评分、Ann Arbor分期及结外侵犯部位数为依据。外周T细胞淋巴瘤预后指数（PIT）纳入年龄、LDH水平、ECOG评分及骨髓侵犯4项指标，更适用于外周T细胞淋巴瘤[21]。在国际关于外周T细胞淋巴瘤研究中，IPI评分对62例EATL亚组人群（EATL Ⅱ型占34％）生存的预测不如PIT，肠道T细胞淋巴瘤患者更适用依据外周T细胞淋巴瘤建立的特异性评分系统[6]。de Baaij等[22]进一步将IPI分层与B症状整合分层，构建EATL预后指数（EPI），其在EATL中对总生存（OS）的预测优于IPI评分与PIT，提示患者临床表型对肠源性T细胞淋巴瘤预后预测的作用不可忽视。但需强调，该研究仅纳入EATL Ⅰ型患者，其与MEITL在流行病学、临床表型及分子特征上并不一致，因此EPI在MEITL中的预后预测作用仍需验证。已有研究识别了MEITL的不良预后因素，包括高龄、体能状态差（ECOG评分>2分）、病变范围广泛（Lugano分期Ⅱ E期及以上）、未能达到完全缓解（CR）、组织学出现非典型特点、TP53突变、MYC高表达等，均与患者生存率下降相关。而约20％病例中出现的B细胞相关抗原表达与较好预后相关[2]。因此，后续应综合患者临床特征（年龄、体能状态）、疾病特征（疾病分期、炎症相关指标）、治疗反应及病理分子特征，并在多中心队列中完成外部验证，从而建立MEITL专有预后模型。

三、治疗策略

MEITL是一种对传统治疗反应欠佳且预后不良的疾病。目前尚无统一的标准治疗方案，临床管理多借鉴其他外周T细胞淋巴瘤的经验，并根据有限的回顾性研究制定策略。总体而言，MEITL的治疗采取综合治疗模式，包括手术、化疗以及造血干细胞移植（HSCT）等措施，此类策略已经存在相对较多的研究总结。而靶向和免疫疗法等新兴探索性研究同样在逐步开展，以下内容将重点综述此类前沿性的治疗策略。附图3（**扫描本文首页二维码查阅**）是基于现有证据与指南建议整合绘制的治疗流程图[23]。

（一）手术

手术在MEITL中既有诊断意义，也有治疗的作用。部分患者以肠穿孔或梗阻起病，紧急外科手术常是诊断和初步治疗的重要步骤。手术切除肿瘤能够去除大部分病灶、解除梗阻并预防再次穿孔，为后续治疗创造条件。

（二）化疗

1. 一线诱导治疗：化疗是MEITL全身治疗的基础。传统的一线方案是以蒽环类药物为基础的CHOP方案。在既往报道中，约37％的患者可以通过CHOP方案达到CR[4]。然而，总体患者的预后仍欠佳，采用CHOP方案治疗的MEITL患者5年生存率仅约20％，而复发率高达80％，多数患者于诊断后1年内死于疾病进展[6]。因此，欧洲肿瘤内科学会（ESMO）-欧洲血液学会（EHA）指南建议初治适合移植的MEITL患者接受非CHOP方案，包括ICE、IVAC、DHAP、DHAX和CHOEP方案，而对于不适合移植的患者，推荐使用减低剂量的ICE、GDP和GEMOX方案[23]。

2. 复发/难治治疗策略：复发/难治MEITL尚无标准救治方案，临床多参照侵袭性外周T细胞淋巴瘤的挽救治疗思路。在真实世界队列中，韩国单中心回顾性研究显示，复发或一线进展后实施的挽救治疗包括DL-ICE、ESHAP、DHAP等多种方案，但CR率只有6.25％（1/16），少数患者在挽救后序贯allo-HSCT可获得较持久缓解。未行移植的患者均死于疾病进展[7]。根据目前有限的证据来看，复发/难治MEITL多采用以铂类/异环磷酰胺/依托泊苷或吉西他滨为骨架的挽救方案，目标是在可接受的不良反应下短暂控制疾病，以顺利桥接HSCT。ESMO-EHA指南将ICE和DHAP方案列为复发/难治MEITL患者的可选方案（ⅢB级）[23]。

盐酸米托蒽醌脂质体作为一种蒽环类药物的脂质体制剂，已在中国获批用于复发/难治外周T细胞淋巴瘤。中国多中心单臂Ⅱ期研究应用米托蒽醌脂质体单药治疗，结果显示客观反应率（ORR）41.7％、CR率23.1％，中位无进展生存（PFS）期8.5个月、中位OS期23.3个月，主要3～4级不良反应为中性粒细胞减少、贫血及血小板减少等血液学不良反应[24]。但上述研究缺乏针对MEITL亚组的数据。目前开展的临床试验正在评估含米托蒽醌脂质体的各种联合方案的疗效与安全性，纳入标准包括MEITL，未来有望明确米托蒽醌脂质体在MEITL中的应用价值。

（三）HSCT

高强度化疗后联合HSCT是改善MEITL患者长期生存的重要策略之一。临床实践中主要采用auto-HSCT和allo-HSCT两种方式，两者在适应证和风险收益方面存在差异。

1. auto-HSCT：对于年轻、初治即缓解的患者，auto-HSCT作为一线巩固方案已被多个中心采用。韩国一项多中心回顾性研究纳入42例MEITL患者，其中16例接受auto-HSCT，单因素分析提示未接受auto-HSCT显著影响预后，提示在对化疗敏感的患者中，获得CR并予以auto-HSCT巩固是改善结局的关键[4]。欧洲一项研究结果分析显示，≤65岁患者中有8例接受auto-HSCT，接受auto-HSCT的患者OS明显优于未接受auto-HSCT的患者（*P*＝0.0067）[2]。日本造血干细胞移植学会基于全国登记系统分析了103例接受HSCT的MEITL患者，其中70例行auto-HSCT，3年OS率为37.5％，3年复发率为63.2％，多因素分析显示移植前未达CR、诊断时LDH升高以及既往化疗线数≥3为OS的独立危险因素，而移植前未达CR是复发的独立预测因素[25]。以上结果提示auto-HSCT的获益高度依赖于移植前的疾病控制及基础身体状况。

2. allo-HSCT：allo-HSCT依赖供者免疫细胞的移植物抗淋巴瘤效应，理论上具有潜在治愈机会，但其应用受限于患者年龄、体能状况、合并症、供者来源及移植相关不良反应等。日本全国移植登记研究中，共有33例MEITL患者接受allo-HSCT，基线特征显示allo-HSCT组非CR状态和移植合并症指数升高的患者比例明显高于auto-HSCT组，提示allo-HSCT多用于病情进展及高危人群；在这33例患者中，3年OS率为23.7％，3年复发率高达78.9％，非复发死亡率约7.1％；多因素分析显示，HSCT合并症指数≥1分及诊断时LDH升高是OS的独立危险因素，而HSCT合并症指数≥1分及移植时ECOG评分为2～4分与高复发风险显著相关[25]。虽然总体复发率偏高，但部分高危患者在allo-HSCT后仍可获得长期生存。受限于基线差异，该研究并未直接比较allo-HSCT与auto-HSCT的优劣，而是强调auto-HSCT更适合作为早期达CR患者的巩固治疗，allo-HSCT则可为部分难治或高危病例提供潜在的治愈机会，但需严格筛选适合人群。

韩国35例单中心队列研究为allo-HSCT的最佳时机提供了重要线索[7]：该研究中，3例获得缓解的患者接受了一线allo-HSCT，随访时3例均维持CR；另有6例复发/难治患者接受挽救性allo-HSCT，其中2例在CR状态下死于感染性并发症，2例复发死亡，2例长期CR生存。生存分析显示，接受allo-HSCT的患者预后明显优于auto-HSCT和未移植患者。

2025年ESMO-EHA指南推荐适合移植的患者在达到CR或PR后优先接受allo-HSCT，而将allo-HSCT前移仍缺乏高质量前瞻性证据支持[23]。现有证据均来自样本有限的回顾性研究。综上，auto-HSCT安全性相对较高，适合作为首次缓解患者的巩固措施，但面临较高的复发风险；allo-HSCT具有潜在治愈作用，尤其适合高度侵袭、对化疗部分敏感或复发/难治的高危患者，但需要权衡移植相关不良反应和感染风险。

（四）靶向治疗

鉴于MEITL具有明确的分子异常谱，靶向治疗被寄予厚望。然而，由于病例较少，目前尚无专门针对MEITL的大型临床试验。基于分子机制的推断和少量病例报道，以下几种潜在靶向及新药治疗思路值得关注。

1. 表观遗传调控药物：MEITL最显著的分子特征是SETD2失活导致的组蛋白H3K36me3缺失，目前尚无能够直接恢复SETD2功能的药物，临床上较可行的策略是利用表观遗传抑制剂改变其下游染色质修饰模式。组蛋白去乙酰化酶（HDAC）抑制剂通过增加组蛋白乙酰化，开放染色质、解除抑癌基因转录抑制，并诱导细胞周期阻滞和凋亡，其已被国际指南纳入复发/难治外周T细胞淋巴瘤的标准选择之一。就MEITL而言，目前证据主要来自病例报道和小样本回顾性研究。Liu等[26]报道2例经根治性切除后接受西达本胺联合化疗的MEITL患者，术后均获得影像学缓解，但分别于11个月和15个月复发；研究认为西达本胺联合方案可在一定程度上延缓复发，但难以根本改变疾病高度侵袭性的特性。Hai等[27]报道了51例T细胞或NK细胞淋巴瘤患者接受含西达本胺维持治疗方案，其中仅有2例MEITL。以上提示HDAC抑制剂具有一定可行性，但仍缺乏针对MEITL的分型分层系统疗效分析。

SETD2失活导致的组蛋白H3K36me3缺失打破了H3K36me3与多梳抑制复合体2（PRC2）/增强子zeste同源物2（EZH2）介导的H3K27me3之间正常的拮抗平衡，使PRC2在增强子区域异常富集H3K27me3，从而沉默一系列抑癌基因和T细胞分化相关基因，维持MEITL细胞高度增殖和抗凋亡状态[28]。抑制EZH2可降低异常H3K27me3水平，恢复抑癌基因表达。在复发/难治外周T细胞淋巴瘤的Ⅰ期扩展队列中，新型EZH2抑制剂SHR2554在28例患者中取得61％的ORR，中位缓解持续时间约12个月。目前针对外周T细胞淋巴瘤的Ⅱ期临床试验（NCT06692452）为评估EZH2抑制剂他泽司他联合CHOP方案的有效性，入组标准明确包括MEITL亚型，试验正在招募中，未公布结果。此外，体外研究显示，长期HDAC抑制剂治疗可诱导H3K27me3上调并产生耐药，而加入EZH2抑制剂可部分逆转这一耐药，协同诱导肿瘤细胞凋亡，为HDAC抑制剂和EZH2抑制剂联合表观遗传治疗提供明确依据[29]。

2. 通路抑制剂：磷脂酰肌醇3-激酶（PI3K）信号通路过度激活在肿瘤发生发展中发挥重要的作用。不同亚型的淋巴瘤中存在不同PI3K亚型的过度激活。在MEITL亚组中，PI3Kα高表达占16.7％（2/12），PI3Kβ阳性占83.3％（10/12），PI3Kδ高表达占50.0％（6/12），PI3Kγ阴性占25.0％（3/12）；在总体队列中PI3Kδ高表达比例最高（66％），且PI3Kδ在各亚型中整体维持较高表达水平，从蛋白表达层面提示MEITL存在PI3K通路相关分子表达基础[30]。在外周T细胞淋巴瘤中的研究中，PI3K通路抑制剂，以PI3Kδ/γ抑制剂度维利塞（duvelisib）为例，PRIMO Ⅱ期试验的结果提示ORR达到48％，CR率为33％，中位PFS期为3.45个月，但不同组织学亚型的获益差异较大，且缺乏针对MEITL亚组的分析[31]。选择性PI3Kδ抑制剂林普利塞（linperlisib）在复发/难治外周T细胞淋巴瘤的Ⅱ期临床试验纳入98例患者，其中包括2例MEITL，结果显示出48％的ORR和30％的CR率[32]。联合策略方面，PI3K抑制剂与表观遗传药物联用是当前较常见的方向。度维利塞联合HDAC抑制剂罗米地辛的Ⅰb/Ⅱa期临床试验纳入了48例可评估的复发/难治外周T细胞淋巴瘤患者，其中包括2例MEITL，结果显示ORR为56％，CR率为44％，中位PFS期为3.5个月，提示探索基于PI3K抑制剂联合策略有望进一步改善MEITL患者的预后[33]。

3. JAK/STAT通路抑制剂：半数以上的MEITL病例存在JAK-STAT信号相关基因的突变激活，提示该通路在肿瘤细胞存活增殖中起关键作用。临床上，JAK1/2抑制剂芦可替尼Ⅱ期研究中设有γδ T细胞淋巴瘤队列，实际纳入的4例患者中包括1例MEITL，结果显示存在JAK/STAT激活证据的病例ORR明显提高[34]。选择性JAK1抑制剂戈利昔替尼在复发/难治外周T细胞淋巴瘤的JACKPOT8研究中同样纳入了少数MEITL患者，整体ORR为44.3％，CR率为24％，但未单独报告MEITL亚组疗效[35]。此外，芦可替尼联合PI3K-δ/γ抑制剂度维利塞的早期临床试验（NCT05010005）正在招募，已将MEITL明确列为可纳入的组织学类型。目前尚无针对MEITL的前瞻性JAK/STAT抑制剂临床试验或系统病例系列报道，但结合MEITL中JAK3/STAT5B高频突变和JAK/STAT抑制剂在其他T细胞淋巴瘤中的抗肿瘤活性，此类药物值得在复发/难治患者中开展临床试验。

4. 其他药物：一些在外周T细胞淋巴瘤中研究的新药也可能对MEITL有效。Ⅱ期单药试验显示BCL2抑制剂维奈克拉在BCL2阳性复发/难治外周T细胞淋巴瘤中疗效受限，提示BCL2抑制剂可以作为联合治疗方案在MEITL病例中进行探索[36]。免疫检查点抑制剂帕博利珠单抗在复发/难治成熟T细胞淋巴瘤中的ORR约30％，部分病例可获得较持久缓解[37]。

（五）细胞治疗

CD7是多数MEITL表达的T细胞抗原，多项早期临床试验显示，靶向CD7的嵌合抗原受体T（CAR-T）细胞疗法在复发/难治急性T淋巴细胞白血病和T细胞淋巴瘤中具有良好的疗效及安全性[38]。一项Ⅰ期临床试验（NCT04928105）纳入5例CD7阳性的复发/难治外周T细胞淋巴瘤，其中包含1例MEITL。在输注后中位34 d评估时，ORR为80％，CR率为60％，不良反应以1～2级细胞因子释放综合征为主，未见神经毒性。2例CR和1例部分缓解的患者进一步接受allo-HSCT巩固，其中1例患者复发并死于移植并发症，其余2例患者维持缓解。未进行allo-HSCT巩固的2例患者均死于疾病进展，提示CAR-T细胞疗法适合作为诱导缓解后桥接移植的策略[39]。其他的靶点如CD30、CD70、CD4、CD5、TRBC1等在外周T细胞淋巴瘤中的临床试验均正在探索[40]–[41]，然而，MEITL的表达差异可能限制其在MEITL患者中的应用，纳入标准需结合组织学检测严格筛选。

除CAR-T细胞外，CAR-NK细胞更易获取以及安全性较高，具现货化与安全性优势，且在靶向CD5、CD7等抗原时不易出现自噬反应。GCC2005是一种靶向CD5的CAR-NK细胞产品，Ⅰ期临床试验（NCT06699771）在7例复发/难治T细胞淋巴瘤中进行探索，初步结果提示其具有良好的安全性和抗肿瘤潜力，但尚未纳入MEITL病例[42]。此外，针对复发/难治T细胞肿瘤的CD5（NCT06909474、NCT05110742）、CD7（NCT07117175）、CD70（NCT06696846）CAR-NK细胞疗法相关临床试验正在进行，但尚无结果报道。

四、疗效评估与随访策略

评估MEITL疗效的核心目标是尽早识别原发难治和缓解后高复发风险的患者。常规评估以临床症状、实验室指标及影像学为基础，必要时结合内镜与病理复评以明确局部病灶控制情况。然而，考虑到MEITL常伴消化道溃疡、穿孔等并发症，重复侵入性检查可能并不适用，因此需引入可重复、动态的监测工具作为补充。

循环肿瘤DNA（ctDNA）作为一种生物标志物不仅能够在部分外周T细胞淋巴瘤患者中检出体细胞突变，而且治疗过程中其负荷的动态下降与复发风险、PFS和OS密切相关。部分患者即便达到影像学CR，若ctDNA突变持续存在，仍可能在随访中复发，提示ctDNA在检测微小残留病（MRD）方面具有优势，MRD状态有望成为复发风险分层的重要因素[43]–[44]。此外，基线ctDNA水平与外周T细胞淋巴瘤患者的预后显著相关[44]，进一步在结外NK/T细胞淋巴瘤人群中进行研究发现，相较于传统预后预测模型，纳入基线ctDNA后具有明显的优势[45]。基于上述结果，未来可探索ctDNA在MEITL中的应用潜力，包括构建新的预后预测模型以及基于ctDNA和影像学的疗效评估框架，用于指导治疗策略的制订和动态调整。

五、总结

综上所述，MEITL是一种罕见且高度侵袭的肠道T细胞淋巴瘤，诊断需结合临床、病理与免疫表型特征及分子学证据。治疗上，目前仍以强化化疗方案联合早期HSCT巩固为主，移植方式和时机有待进一步研究，患者总体疗效及预后仍较差。未来应积极进行临床试验以验证MEITL患者应用新型靶向免疫治疗的获益情况。

## Supplementary Material


