# Supplementary figures and images for: Current status and progress in the diagnosis and treatment of monomorphic epitheliotropic intestinal T-cell lymphoma
Source: Zhonghua Xue Ye Xue Za Zhi. 2026 May;47(5):500–6. [Article in Chinese] doi: 10.3760/cma.j.cn121090-20251128-00557 (PMC13416541; doi:10.3760/cma.j.cn121090-20251128-00557)

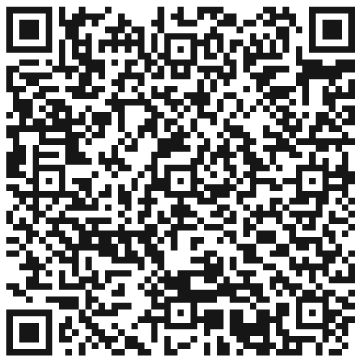

Supplement: Supplementary file 1 [file cjh-47-05-500-g001.tif]
